# Linkage of HIV treatment and population-based surveillance records in rural South Africa: the AHRI Unified Data Platform (AUDP)

**DOI:** 10.1186/s13690-026-01849-8

**Published:** 2026-02-07

**Authors:** Dickman Gareta, Evelyn Lauren, Khumbo Shumba, Cornelius Nattey, Matthew P. Fox, Koleka Mlisana, Matthias Egger, Dorina Onoya, Kobus Herbst, Jacob Bor

**Affiliations:** 1 Africa Health Research Institute, Somkhele campus, R618 enroute to Hlabisa, Mtubatuba, Durban, KwaZulu-Natal 3935 South Africa; 2 Institute of Social and Preventive Medicine, University of Bern, Bern, Switzerland; 3 Graduate School for Health Sciences, University of Bern, Bern, Switzerland; 4 School of Laboratory Medicine and Medical Sciences, University of KwaZulu Natal, Durban, South Africa; 5 Department of Biostatistics, Boston University School of Public Health, Boston, MA USA; 6 Health Economics and Epidemiology Research Office, Faculty of Health Sciences, University of the Witwatersrand, Johannesburg, South Africa; 7 Department of Global Health, Boston University School of Public Health, Boston, MA USA; 8 Department of Epidemiology, Boston University School of Public Health, Boston, MA USA; 9 DSTI-SAMRC South African Population Research Infrastructure Network (SAPRIN), Durban, South Africa; 10 National Health Laboratory Service, Centre for HIV and STIs, National Institute for Communicable Diseases, Johannesburg, South Africa; 11 Centre for Infectious Disease Epidemiology and Research, Faculty of Health Sciences, University of Cape Town, Cape Town, South Africa; 12 Population Health Sciences, Bristol Medical School, University of Bristol, Bristol, UK; 13 Department of Infectious Diseases and Hospital Epidemiology, University Hospital Zurich, University of Zurich, Zurich, Switzerland

**Keywords:** South africa, Probabilistic record linkage, HIV care cascade, HIV epidemiology, HIV treatment outcomes

## Abstract

**Background:**

Integrating HIV clinical records with population-based surveillance data allows the study of health care seeking behaviours, access to care, and predictors of patient outcomes. We implemented a graph-based record linkage algorithm to deduplicate and link HIV clinical and population-based surveillance records in an HIV-endemic setting in rural South Africa.

**Methods:**

We linked four data sources to create the Africa Health Research Institute (AHRI) Unified Data Platform: AHRI’s Health and Demographic Surveillance System (HDSS), AHRI Clinic and Hospital Information System (AHRILink), National Health Laboratory Service (NHLS), and Three Integrated Electronic Registers (TIER.Net) HIV care and treatment records. HDSS data were collected between January 1, 2000, and July 31, 2024, through repeated household surveys of over 140,000 individuals. Clinical and laboratory data were obtained for one hospital and 17 clinics in Hlabisa, KwaZulu-Natal, covering the HDSS surveillance area. We implemented a probabilistic record linkage algorithm trained and validated on a subset of records with national identity numbers. We assessed linkage accuracy, computed descriptive statistics for the linked database, and estimated the HIV care cascade for this population.

**Results:**

A total of 986,832 records were successfully linked across the four databases, achieving a sensitivity of 92.7% and a positive predictive value of 96.5% (F-score=0.95). The average number of records (standard deviation (SD)) in TIER.Net, HDSS, AHRILink and NHLS were 1.18 (0.44),1.05 (0.23),1.13 (0.40), and 5.21 (4.24), respectively. The linked data indicated that 12,293 HDSS resident adults (≥15 years) were living with HIV at some point during the 2022 and 2024 surveillance rounds. Of these, 10,622 (86.4%) had ever sought HIV care in the public sector, of whom 10,492 (98.8%) had ever started ART and 7,065 (66.5%) were currently on ART, of whom 6,301 (89.2%) were virally suppressed(viral load<200 copies/mL).

**Conclusion:**

HIV care and population surveillance records from four data sources were deduplicated and linked with high accuracy, revealing persistent gaps in retention in care and viral suppression in an HIV-endemic region in rural South Africa. The AHRI Unified Data Platform offers the potential to deepen our understanding of HIV epidemiology in a well-described population and to improve services for HIV.

**Trial registration:**

Not applicable.

**Supplementary Information:**

The online version contains supplementary material available at 10.1186/s13690-026-01849-8.

**Table:** 

Text box 1. Contributions to the literature
• This study establishes a replicable framework for integrating heterogeneous health and demographic data sources (surveillance, clinical systems, laboratory records, and HIV databases) to advance HIV epidemiological research and improve health service delivery.
• Demonstrates the successful application of a graph-based probabilistic record linkage algorithm, achieving high accuracy, and provides a methodological benchmark for deduplication and linkage in resource-limited contexts.
• Provides empirical evidence on gaps in the HIV care cascade—particularly retention and viral suppression—despite high ART coverage rates, informing targeted interventions and policy.
• Offers a comprehensive, longitudinal dataset enabling robust analyses of HIV care-seeking behaviors, treatment uptake, and retention.

## Background

As 2030 approaches, reliable HIV programmatic data are essential for monitoring progress toward global treatment goals. The UNAIDS 95-95-95 targets aim for 95% of all people living with HIV (PLHIV) to know their status, 95% of those diagnosed with HIV to receive sustained antiretroviral therapy (ART), and 95% of those on treatment to achieve viral suppression. Yet, many HIV programs - especially those from resource-limited settings - lack robust data management systems that track individuals as they navigate through the healthcare system from HIV diagnosis to viral suppression [1, 2], let al.one to track people who have disengaged from care.

Linkage of health records with population-representative surveys has become a mainstay of epidemiological research [3], but is little used in the field of HIV. Linking HIV treatment records with population-based surveillance data could provide a powerful research platform to study healthcare access [4], HIV disease progression and treatment outcomes [5, 6], social determinants of HIV risk and care [7, 8], impacts of new clinical guidelines [9], and the broader health, economic, and social impacts of HIV care [10–12].

The Africa Health Research Institute (AHRI)’s Health and Demographic Surveillance System (HDSS) has collected information on the demographic, socioeconomic, and health status of persons residing in a geographically defined area of rural KwaZulu-Natal, South Africa, since 2000 [13]. HIV biomarkers have been collected since 2003, enabling the identification of people living with HIV (PLHIV). PLHIV residing in the HDSS are served by 17 public sector primary health facilities that provide HIV care and treatment for free at the point of service. HIV clinical data are managed and stored in the Three Integrated Electronic Registers (TIER.Net) HIV treatment database [14]. HIV care is guided by laboratory testing to determine whether patients are at risk for treatment complications and to monitor treatment. Laboratory records are stored centrally in the National Health Laboratory Service (NHLS) database. Additionally, AHRI maintains a database of clinic visits and hospitalizations for both HIV-related and non-HIV-related care, enabling assessment of HIV comorbidities. To date, these four databases have not been linked.

We implemented a graph-based probabilistic record linkage algorithm to deduplicate and link the HIV treatment, laboratory, HDSS, clinic visit and hospital admission records, creating the AHRI Unified Data Platform (AUDP). The AUDP has potential to generate rigorous evidence on high-priority areas to help achieve the goal of ending the HIV epidemic by 2030.

## Methods

### Setting

The data are from Hlabisa subdistrict, northern KwaZulu-Natal, South Africa. The subdistrict has 17 primary healthcare facilities serving a population of over 600,000 people. These facilities are run by the Department of Health and provide outpatient care, including chronic HIV management and care. All laboratory tests performed in these healthcare facilities are sent to the NHLS for processing. Prior research from AHRI has shown that the rollout of HIV treatment has increased life expectancy by over a decade and led to significant economic benefits for PLHIV and their households [10]. Nevertheless, the area continues to experience a very high burden of HIV, with an incidence of approximately 3.4 per 100 person-years [15], placing it among the highest incidence settings globally.

### Data sources

#### AHRI health and demographic surveillance system

The AHRI has run a HDSS since 2000, which currently covers all households in an 845km^2^ area nested within Hlabisa subdistrict. The HDSS has been extensively described elsewhere [13]. Briefly, data on vital events such as births, deaths and migrations are collected through annual household surveys for a population of over 140,000 individuals from 20,000 households [13]. All households are geolocated, which allows analysis of spatial relationships. A standardized World Health Organization verbal autopsy questionnaire is administered for all deaths to ascertain the causes of death [16]. HIV dried blood spots, sexual behaviour and general health data are annually collected from eligible individuals aged 15 years and above to understand the transmission patterns, drivers and burden of HIV disease in the community.

#### TIER.Net HIV treatment database

All South African government health facilities use an electronic HIV treatment monitoring system known as TIER.Net [14]. Most health facilities use a non-networked version of the system due to limited connectivity and because of its ease of installation and maintenance. The TIER.Net database contains information on patient demographics such as patient first name, surname, date of birth, gender, South African identity (ID) number and health facility name. Through a memorandum of agreement between AHRI and South Africa’s Department of Health, AHRI had access to demographic information and test results until May 2020 – before the Protection of Personal Information (POPI) Act came into effect in July 2020. This access was intended to facilitate record linkage with the HDSS database.

The TIER.Net records information on clinic visits, ART dispensing records and laboratory test results for patients on ART. The system assigns each patient a universally unique identifier. However, these identifiers are not always unique in practice. Duplicate patient identifiers can arise when patient files cannot be retrieved and new identifiers are issued, when patients transfer between health facilities, or when patients re-engage in care after discontinuing ART and their original file cannot be traced. Laboratory results from the NHLS are manually entered into the TIER.Net system from patient files by experienced data clerks. However, errors in recording laboratory result data are common. In addition, some laboratory results are misfiled or lost before they can be captured. As we have shown previously, the majority of the laboratory results in the TIER.Net do not have specimen identifiers, making it difficult to link HIV treatment data with laboratory databases [17].

The database contains patients who initiated ART in the 17 health facilities in the Hlabisa subdistrict between 2004 and 2020, with longitudinal data on ART clinic visits for those patients were available up to 2024.

#### NHLS laboratory database

The NHLS is the sole provider of diagnostic pathology services for the public health sector in South Africa and conducts all CD4 counts, HIV viral loads, and other monitoring tests in South Africa’s HIV treatment programme. Each record in the NHLS database represents a laboratory test result. NHLS was implemented in KwaZulu-Natal Province in 2010, and the AHRI received laboratory test results for the 17 primary health facilities in the Hlabisa subdistrict through 30 September 2022. The database contains information on patients’ demographics such as patient first name, surname, date of birth, gender, South African national ID number, along with test types, result values, and the date samples were collected. Since the present study focused on HIV, only CD4 counts, HIV viral loads, and HIV diagnostic tests, and treatment workup test results were included.

#### AHRILink hospital and clinic information system

AHRI has set up a hospital and clinic information system known as AHRILink at a local district hospital and the 11 public health facilities located within the surveillance area. Since 2010 a trained nurse and two clinical research assistants have prospectively captured data for each admission of HDSS resident and non-resident patients at the hospital. The data include patient demographic information, admissions, discharges and death diagnoses, which are coded according to the International Classification of Diseases,10th Revision (ICD-10). Additionally, AHRI clinical research assistants in the 11 primary care facilities capture data for all patients visiting the clinics. The data include demographic information and reasons for health facility attendance, including among other reasons HIV testing, ART initiation and follow-up, minor ailment, hypertension, diabetes, and mental health. Demographic data of individuals from the HDSS area are regularly updated in the AHRILink database to facilitate linkage.

### Record linkage

**Fig. 1:**
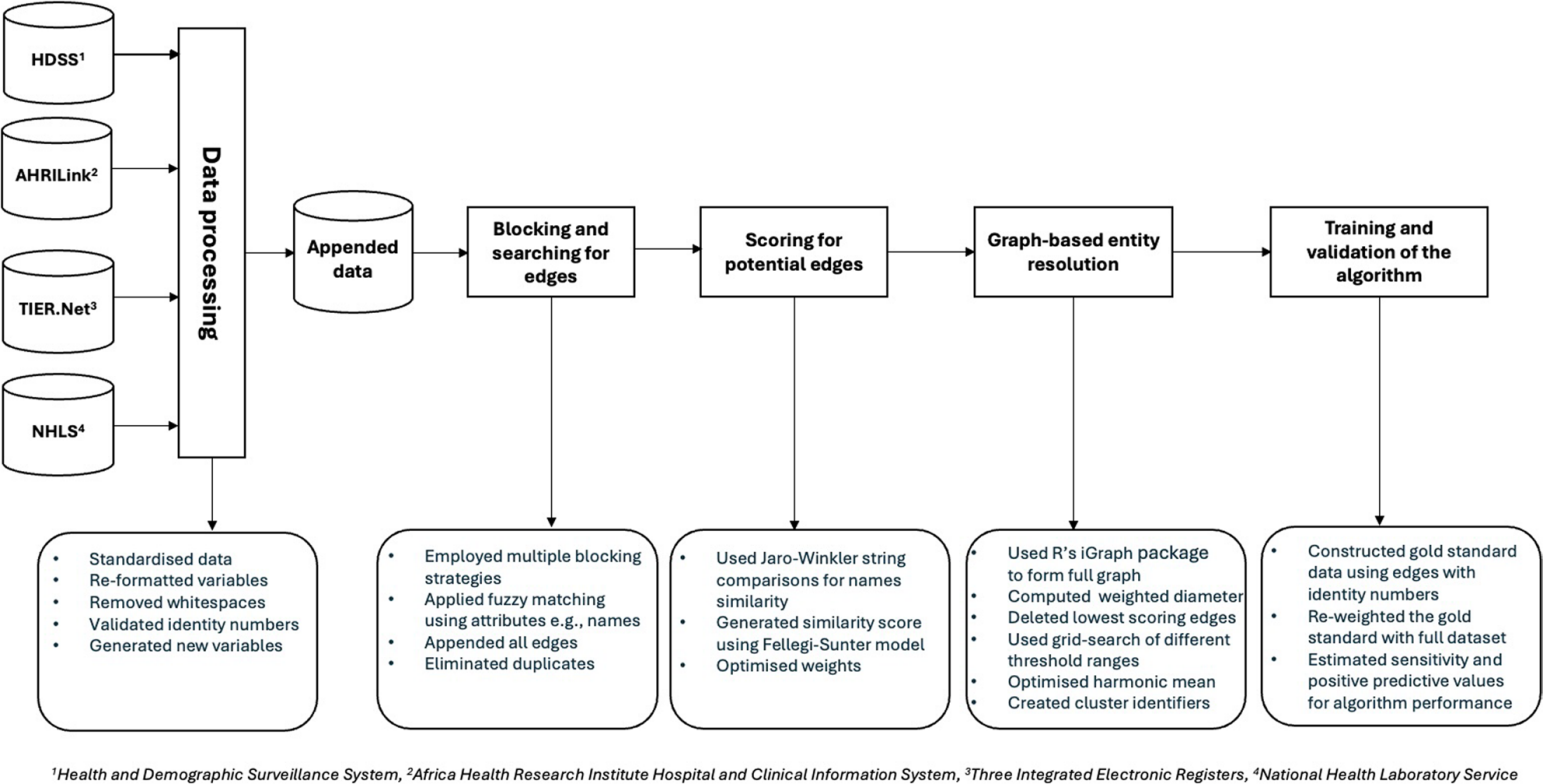
Data processing, linkage and deduplication processes

We adapted a graph-based probabilistic record linkage algorithm developed for national deduplication of the national NHLS database [18, 19], which we used to simultaneously deduplicate and link the four databases above. The record linkage approach involves four steps (Fig. 1): (1) preprocessing of the data to ensure each data point has common identifying variables; (2) searching for potential matches, using blocking strategies to reduce the search space; (3) scoring of potential matches; and (4) entity resolution, in which clusters of records corresponding to unique persons are identified.

#### Preprocessing

Preprocessing of the data involved data cleaning, standardization and removal of blank spaces due to typographical errors in recording the data. We standardized the four data sources to ensure that all the variables had the same format (Fig. 1). Records in each of the four databases were associated with a first name, surname, date of birth, gender, South African ID number, name of health facility, and source database. There were 986,832 records across the four data sources (Table 1).

**Table 1:** Data source characteristics and completeness before deduplication and linkage

Characteristics of each database	TIER.Net		NHLS	HDSS	AHRILink		Total
Data description	HIV clinical records		HIV laboratory records	Household and individual records		Individual clinic visit records	n/a^**δ**^
Number of records in each database before deduplication	75,801		474,082^a^	258,391	178,558		986,832
Number of unique individuals (deduplicated)	64,140		97,757^b^	246,945	158,440		414,007
Number of records per individual							
Mean (SD)	1.18 (0.44)		5.21(4.24)	1.05 (0.23)	1.13 (0.40)		n/a
Median (IQR)	1(1–1)		4(2–7)	1(1–1)	1(1–1)		n/a
Range	1–7		1–40	1–6	1–9		n/a
Type of records	Patients seeking HIV care		Lab specimens used for HIV care	Individuals in the HDSS	Patients seeking care for HIV or other conditions		n/a
Years of data	2004–2024		2010–2022	2000–2024	2017–2024		n/a
Geographic coverage	17 clinics, 1 hospital in Hlabisa subdistrict		17 clinics, 1 hospital in Hlabisa subdistrict	845 km^2^ HDSS nested in Hlabisa subdistrict	10 clinics in the HDSS, nested in HDSS area and 1 hospital within Hlabisa subdistrict		n/a
Percent HIV+	100%		100%	32% ^**α**^	28%^**β**^		
Completeness of identifiers used for matching							
		%	%	%	%		%
Surname	100		100	100	100		100
First name	100		100	100	100		100
Gender	100		96	100	100		100
South Africa ID number	71		27	41	21		33
Date of birth	100		98	99	99		99
Health facility	100		100	100	100		100

^α^ Estimated based on population-based biomarker surveillance in the AHRI HDSS, ^β^ the percent of patients who are living with HIV ,^δ^ are not applicable, ^a^ – Specimen episodes, ^b^ – individuals, SD – standard deviation, IQR – interquartile range

The NHLS records were composed of unique specimen IDs for the CD4 count, viral load and treatment workup test results collected between 2010 and 2022. TIER.Net records corresponded to patients who started HIV treatment between 2004 and 2020 and were longitudinally followed up until 2024. AHRI HDSS records were individuals who ever registered in the HDSS between 2000 and 2024 and AHRILink records were individuals who were ever admitted at the hospital between 2010 and 2024 or who ever visited a health facility for any reason between 2017 and 2024.

#### Search for potential matches

We employed 12 overlapping blocking strategies on combinations of the following attributes: first four letters of the first name, first four letters of the surname, last four letters of first name, last four letters of surname, gender, date of birth, day of birth, month of birth and year of birth (Supplementary Table S1). For each blocking strategy, the combined dataset was joined with itself on the blocking attributes, and candidate record pairs matching on these attributes were set aside for scoring. Candidate matches produced from these blocking strategies were appended together and deduplicated to create a list of potential matches for scoring.

#### Scoring for similarity

We followed an adapted Fellegi-Sunter model [20, 21] to score the similarity of each record pair across five domains: first name, last name, DOB, gender and facility. National IDs, available for a minority of record pairs, were used to train and validate the model, and where available we deferred to National IDs to determine match/non-match. There are two types of marginal probabilities that are used in the Fellegi-Sunter model: The *m* probability is defined as the probability that an attribute matches given that the candidate record pair matches. The *u* probability is the probability that an attribute matches given that the candidate record pair does not match. Using these marginal probabilities, we computed a similarity score for each attribute, defined as the logarithm of the base 2 (log_2_) of the ratio of the *m* and *u* probabilities if the records agreed on that attribute; and the log_2_ of the ratio of (1-m) and (1-u) if the records disagreed on that attribute. The *m* probabilities for the attributes were computed based on representative training data from a prior validation study [20]. The u probabilities were calculated as the maximum of the two probabilities at which a specific attribute value appeared in the database. Jaro–Winkler scores [18] ranging between 0 and 1 were used to summarize similarities of first names and surnames, and these scores were used to generate a weighted average of agreement and disagreement scores [21].

After computing the similarity scores for each attribute, we aggregated these attribute-specific scores into a total similarity score, implicitly weighting the scores by their discriminative power or importance. Thus, a match on a rare first name received a large positive score, as this was unlikely to occur by chance, whereas a mismatch on gender received a small negative score, as this was more likely to occur by chance.

#### Graph-based entity resolution

Individuals may be observed across each of the four databases and observed multiple times within each one. Our goal was to identify the cluster of records corresponding to each individual. We used a graph-based entity resolution strategy, conceptualizing the records to be linked as vertices or nodes and the similarity scores between records as edges. Clusters too large to represent a single patient were broken up using a variable threshold method developed by Bor et al. [18]. The variable threshold method was optimized using South African national ID numbers as training data, as described below. We used the iGraph package in R to create networks.

**Fig. 2:**
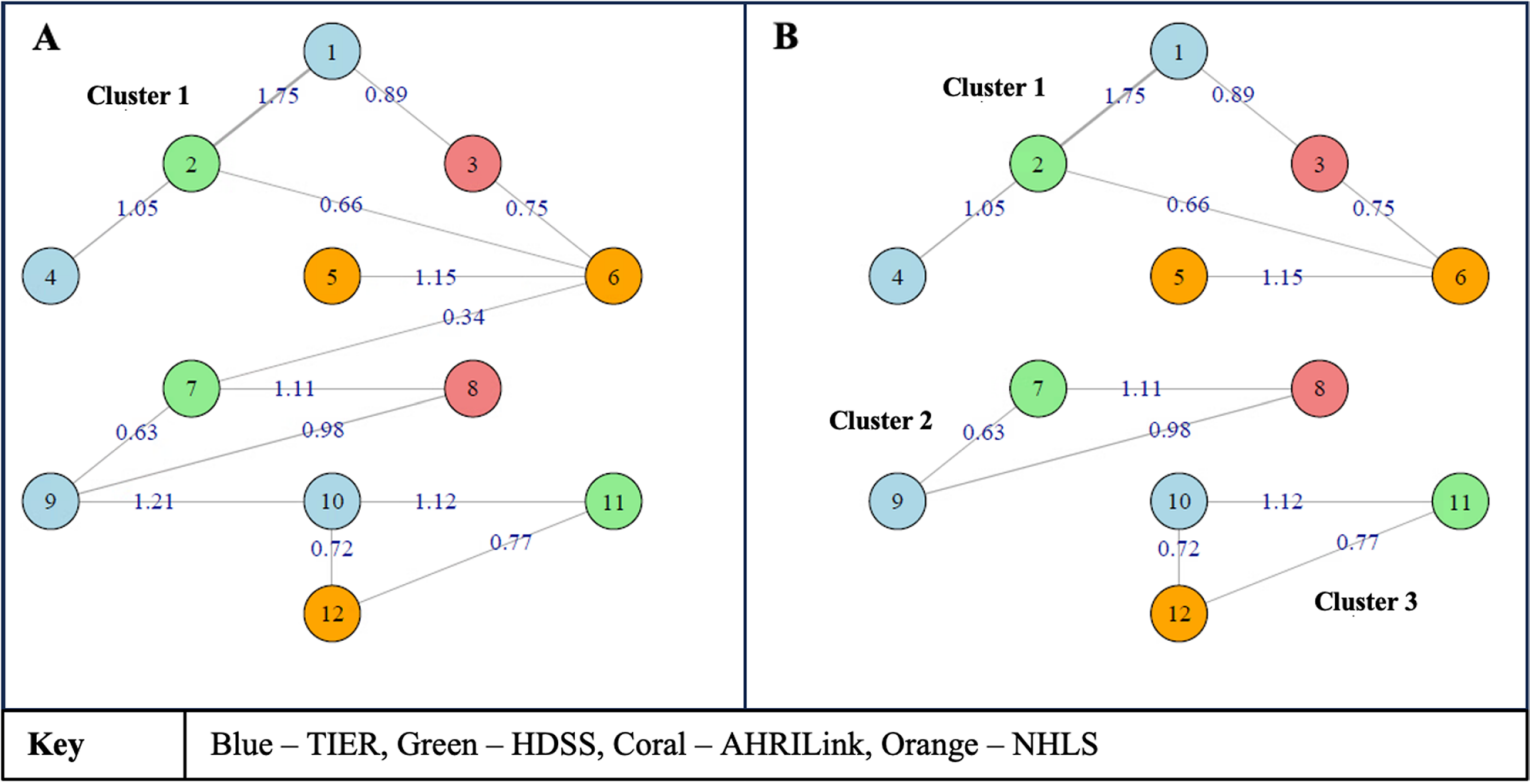
Illustration of the graph-based record linkage where a large cluster (Panel **A**) is iteratively broken down into three component clusters (Panel **B**) until each cluster’s diameter fell below a threshold

We converted the total similarity scores into a quasi-probability measure *p* (where 0 ≤ *p* ≤ 1), by fitting a logistic regression model of National IDs(match/non-match) on the total similarity score. We then converted this probability into a distance measure d, defined as: $d=-log\left(p\right)$, which was used to compute the weighted distance (or diameter) in the clustering process (Fig. 2A). The weighted diameter of a cluster is the distance between its two most dissimilar (i.e., farthest) nodes. To determine the optimal threshold for breaking up clusters, we performed a grid search over a range of weighted diameter thresholds between 0 and 1. For each candidate threshold α, we examined whether the maximum distance in each cluster exceeded *-log(α)*. If it did, we iteratively removed the edge with the lowest match probability until each cluster’s diameter fell below the threshold (i.e., all shortest paths between nodes were less than -log(α)) (Fig. 2B).

### Construction of the quasi-gold standard data

We constructed quasi-gold standard data using records containing South Africa ID numbers. The Department of Home Affairs issues South African ID numbers to South African citizens and permanent residency holders who are 16 years or older. The South African ID number is a 13-digit number of the following format YYMMDDSSSSCAZ where YYMMDD denotes the date of birth, SSSS denotes gender, C denotes citizenship status and Z is a checksum digit to check if the number sequence is accurate. We validated the South African ID numbers in the master data using the Luhn algorithm, a simple checksum formula used to validate identification numbers [22]. All invalid ID numbers were coded as missing. We further validated the South African ID numbers by ensuring that the date of birth in the ID matched the recorded date of birth.

### Training and validation of the algorithm

The performance of the record linkage algorithm was assessed using positive predictive value (PPV), sensitivity and F-score, which are defined as follows:

$$\mathrm{PPV}\;=\;\frac{Number\;of\;record\;pairs\;matched\;by\;cluster\;ID\;and\;national\;ID\;}{Number\;of\;record\;pairs\;matched\;by\;cluster\;ID}\;$$

$$\mathrm{Sensitivity}\;=\frac{\mathit N\mathit u\mathit m\mathit b\mathit e\mathit r\mathit\;\mathit o\mathit f\mathit\;\mathit r\mathit e\mathit c\mathit o\mathit r\mathit d\mathit\;\mathit p\mathit a\mathit i\mathit r\mathit s\mathit\;\mathit m\mathit a\mathit t\mathit c\mathit h\mathit e\mathit d\mathit\;\mathit b\mathit y\mathit\;\mathit c\mathit l\mathit u\mathit s\mathit t\mathit e\mathit r\mathit\;\mathit I\mathit D\mathit\;\mathit a\mathit n\mathit d\mathit\;\mathit n\mathit a\mathit t\mathit i\mathit o\mathit n\mathit a\mathit l\mathit\;\mathit I\mathit D}{\mathit N\mathit u\mathit m\mathit b\mathit e\mathit r\mathit\;\mathit o\mathit f\mathit\;\mathit r\mathit e\mathit c\mathit o\mathit r\mathit d\mathit\;\mathit p\mathit a\mathit i\mathit r\mathit s\mathit\;\mathit m\mathit a\mathit t\mathit c\mathit h\mathit e\mathit d\mathit\;\mathit b\mathit y\mathit\;\mathit n\mathit a\mathit t\mathit i\mathit o\mathit n\mathit a\mathit l\mathit\;\mathit I\mathit D}$$

$$\mathrm F-\mathrm{score}\;=\;2^{\ast\;}{\textstyle\frac{Sensitivity^{\mathit\ast}PPV}{Sensitivity+PPV}}$$

We defined PPV (also known as precision) as the proportion of algorithm-designated matches (i.e., matches using the variable threshold method) that are true matches, whereas sensitivity (also known as recall) denotes the proportion of true matches that are designated as matches by the algorithm. As we noted in previous work, specificity and negative predictive value are not useful evaluation markers in situations where the number of true non-matches is high [17].

To calculate PPV and sensitivity, we included both the edges we explicitly identified and scored, as well as implicit edges formed through transitivity (e.g., if A links to B and B links to C, then there is an implicit edge between A and C). Because only record pairs with national IDs were used to calculate PPV and sensitivity, we applied weights to adjust for bias, ensuring that these measures reflect the values that would have been obtained if all record pairs (with and without national IDs) had been included [23]. We computed the F-score measure as a harmonic mean of sensitivity and PPV.

## Results

### Data source completeness

All four data sources had complete records for first names, surnames and facility (Table 1). The four data sources had nearly complete data for first name, surname, gender, date of birth and healthy facility.

**Fig. 3:**
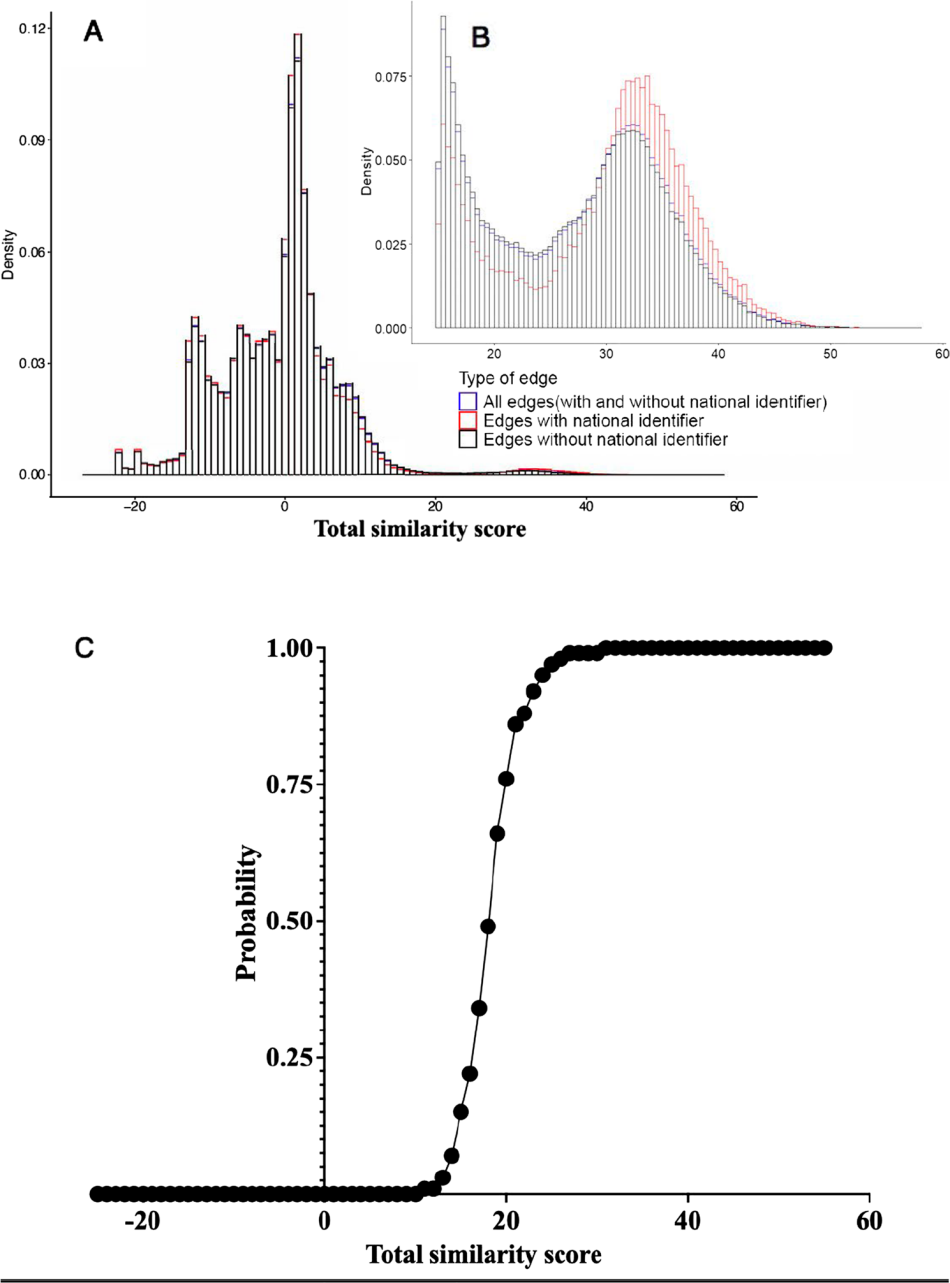
**A** Distribution of total similarity scores for all edges, edges with South African identifiers and edges with no South African identifiers, (**B**) distribution of total similarity scores above 15 for all edges, edges with South African identifiers and edges with no South African identifiers, (**C**) probability that national identifiers match given total similarity score

Majority of the records in TIER.Net had South African IDs relative to the other data sources (Table 1). Overall, one out of three records had a valid South African ID number. We created a master dataset comprising of 268,348,185 edges, of which 10.8% had valid South African ID number. The distribution of total similarity scores was bimodal, consistent with the hypothesized data-generating process in which scored edges are a mixture of two distributions representing the scores for true matches and the scores for true non-matches (Fig. 3A). Most record pairs were true non-matches, with low similarity scores. However, a group of record pairs that were true matches had higher similarity scores and are shown as a distinct hump to the right side of the distribution (Fig. 3B). A well-performing total similarity score yields distinct distributions such that a threshold cut-off can be easily established. Of note, the level of uncertainty of linked records was high for similarity scores between 18 and 30 (Fig. 3C).

### Assessment of algorithm performance

After the sensitivities and PPVs for various weighted distance thresholds were computed, the best weighted distance was estimated to be 0.6 (F-score = 0.95), with an adjusted sensitivity of 92.7% and adjusted PPV of 96.5%. (Table 2).

**Table 2:** Performance of the linkage at different clustering thresholds

Threshold*	0.1	0.2	0.3	0.4	0.5	0.6	0.7	0.8	0.9
Number of patient ID clusters	133,579	143,384	150,385	156,392	161,824	167,171	172,897	179,493	189,298
Sensitivity (unadjusted)	0.97	0.97	0.96	0.95	0.95	0.94	0.93	0.91	0.89
PPV (unadjusted)	0.86	0.90	0.92	0.94	0.96	0.97	0.98	0.98	0.99
F-score (unadjusted)	0.91	0.93	0.94	0.95	0.95	0.95	0.95	0.95	0.94
Sensitivity (bias-corrected)	0.97	0.96	0.96	0.95	0.94	0.93	0.91	0.90	0.87
PPV (bias-corrected)	0.84	0.88	0.91	0.93	0.95	0.97	0.97	0.98	0.99
F-score (bias-corrected)	0.90	0.92	0.93	0.94	0.94	0.95	0.94	0.94	0.92

*The table shows the performance at nine different sensitivity levels for the graph-based entity resolution step of the linkage. In the algorithm, we computed the weighted distance between the farthest two points in each cluster as a measure of the likelihood that the cluster combined more than one unique individual. Thresholds are the values below which a cluster was deemed too large to be a single patient

This means that 92.7% of the true matches were designated as matches by the algorithm and that 96.5% of the algorithm-designated matches were true matches. We noted that the weighted measures, i.e., the bias-corrected measures had lower F scores compared to the unweighted measures, because edges without South African ID numbers had more uncertainty in the middle range while edges with South African ID were less likely to have much uncertainty (either very high scores or very low scores) (Fig. 3C**)**.

After deduplication and linkage, 414,007 unique individuals were identified (Table 1). The average number of records (standard deviation (SD)) in TIER.Net, HDSS, AHRILink and NHLS were 1.18 (0.44),1.05 (0.23),1.13 (0.40) and 5.21 (4.24) respectively.

After deduplicating and linking the four databases, we conducted descriptive analyses to assess the extent of overlap and basic characteristics of the resulting database. AHRI’s Unified Data Platform can be used to extract sub-cohorts with different inclusion criteria. For example, an HIV Care and Treatment Cohort can be constructed, including all persons receiving HIV care and treatment services (TIER.Net), with complete linked laboratory data and additional clinical and population-based data available for patients residing in the HDSS or receiving care at 11 of.

the 17 clinics. Alternatively, a population cohort can be constructed for all persons residing in the HDSS, including people who are HIV+, HIV-, and of unknown HIV status.

Table 3 shows the characteristics of the HIV Care and Treatment Cohort between 2004 and 2020, as recorded in the TIER.Net. Of the 64,140 unique individuals in TIER.Net, 58,815 (91.7%) were linked to NHLS, 25,175 (39.3%) were linked to HDSS, and 27,798 (43.3%) were linked to AHRILink. Based on the vital status data from TIER.Net, HDSS and AHRILink (hospital admissions), individual outcomes at the last observation were as follows: 30,087 (46.9%) were alive and on treatment, 6,958 (10.9%) died, 14,336 (22.4%) were lost to follow up (include individuals with ≥365 days since their last documented HIV treatment visit or scheduled ART visit) and 12,759 (19.9%) were transferred out. Additionally, 16,074 (25.1%) of the individuals in TIER.Net had at least one hospital admission. Based on integrated laboratory data from NHLS and the TIER.Net, 55,889 (87.1%) of patients in TIER.Net had at least one recorded viral load, of which 76.5% were < 200 copies/mL.

**Table 3:** HIV care and treatment cohort: patients who started ART between 2004 and 2020

Characteristic	HIV treatment and care cohort (*n* = 64,140)		
		*n*	%
Gender ^α^	Female	43,519	67.9
	Male	20,621	32.2
Age at ART initiation ^α^	0–9	2,655	4.1
	10–19	3,490	5.4
	20–29	21,289	33.2
	30–39	21,180	32.0
	40–49	9,798	15.3
	50–59	4,163	6.5
	60+	1,323	2.1
	Unknown	242	< 1
Patient outcome at last visit ^β^	Alive and on treatment	32,087	50.0
	Died	7,320	11.41
	Lost to follow up	13,737	21.41
	Transfer out	10,854	16.9
Status at ART initiation ^α^	ART naïve	48,227	75.2
	Transfer-in	15,682	24.5
	Unknown	231	< 1
Latest ART regimen ^α^			
First line	Dolutegravir-based	36,082	56.3
	Efavirenz-based	25,572	39.9
	Other first line	1,484	2.3
Second line	Dolutegravir-based	236	0.4
	Lopinavir-based	610	1.0
	Other second line	38	0.1
Third line	Dolutegravir-based	6	< 1
	Ritonavir-based	9	< 1
	Unknown	103	0.2
Last recorded viral load ^δ^	< 200	49,089	76.5
	200–1000	1,724	2.7
	> 1000	5,076	7.9
	Unknown	8,251	12.9
CD4 count at ART initiation ^δ^	≤200	21,239	33.1
	> 200	32,151	51.7
	Unknown	9,750	15.7
Tuberculosis status	No	42,259	65.9
	Yes	6,811	10.6
	Unknown	15,070	23.5
WHO stage^Ψ^	1	31,724	49.5
	2	10,196	15.9
	3	10,515	16.4
	4	1,509	2.4
	Unknown	10,196	15.9
Hospital admission episodes ^ε^	0	48,066	74.9
	1	10,888	17.0
	2	3,477	5.4
	≥3	1,709	2.7
Clinic visit ^ε^ (Median, IQR)		18(8–29)	

^α^From TIER.Net, ^β^From TIER.Net, HDSS and AHRILink (hospital admissions), ^δ^From TIER.Net and NHLS and ^ε^From AHRILink, ^Ψ^WHO – World Health Organization

Table 4 presents Population Cohort characteristics, including individuals ever observed in the AHRI HDSS between 2000 and 2024. Among the 246,945 unique individuals with data in the HDSS, 43,325 (17.5%) were identified as HIV positive based on at least one of the following: a positive HIV DBS test result from the HDSS, a patient record in TIER.Net, an HIV-related clinic visit or hospital admission in AHRILink, or a CD4 count or HIV viral load result from NHLS. Of these PLWH, 31,051 (71.9%) had a record in TIER.Net, NHLS, or AHRILink (clinic visits and hospital admission), indicating that they had accessed HIV care, and of those, 25,175 (81.1%) had a record of having initiated ART in TIER.Net. For those who attended clinics, the median number of clinic visits was 5 (IQR 2–15).

**Table 4:** Population cohort: individuals residing in the AHRI HDSS, 2000–2024

Characteristic	Population cohort (*n* = 246,945)		
		*n*	%
Gender ^σ^	Female	132,028	53.5
	Male	114,914	46.5
	Missing	3	< 1
Age at last visit date ^σ^	0–9	40,013	16.2
	10–19	48,956	19.8
	20–29	50,721	20.5
	30–39	43,717	17.7
	40–49	25,089	10.2
	50–59	14,555	5.9
	60+	21,893	8.9
	Missing	2001	< 1
Surveillance status ^σ^	Alive	152,713	61.8
	Membership ended	37,350	15.1
	Outmigration	10,886	4.4
	Other	19,093	7.7
	Died	26,903	10.9
	Of which, cause of death		
	HIV/AIDS and TB	7,567	29.0
	CMPN	2,013	7.4
	NCD	3,338	12.3
	Injuries	1,503	5.6
	Unknown	12,482	46.7
Location type ^σ^	Rural	162,445	65.8
	Peri-urban	67,163	27.2
	Urban	16,269	6.6
	Missing	1,068	< 1
Employment status ^σ^	Yes	38,590	25.9
	No	110,671	74.2
Education ^σ^	None	14,401	5.8
	Primary	47,838	19.4
	Secondary	96,605	39.1
	Tertiary	25,727	10.4
	Unknown	62,374	25.3
Wealth quintile ^σ^	Poorest	68,492	27.7
	Poor	33,775	13.7
	Medium	35,920	14.6
	Rich	44,873	18.2
	Richest	45,761	18.5
	Missing	18,124	7.3
HIV status ^λ^	Unknown	153,305	62.1
	Negative	50,314	20.4
	Positive	43,326	17.5
	Of those who are HIV-Positive		
	Ever accessed care^α,λ,ε^	31,049	71.7
	Ever started ART^α^	25,175	58.1
Hospital admission episodes ^ε^	0	224,570	90.9
	1	17,080	6.9
	2	3,834	1.6
	≥3	1,461	< 1
Clinic visit ^ε^ (Median, IQR)		5(2–15)	

^σ^From HDSS, ^α^From TIER.Net, ^λ^NHLS, and ^ε^From AHRILink

### Progression through the HIV care cascade

**Fig. 4:**
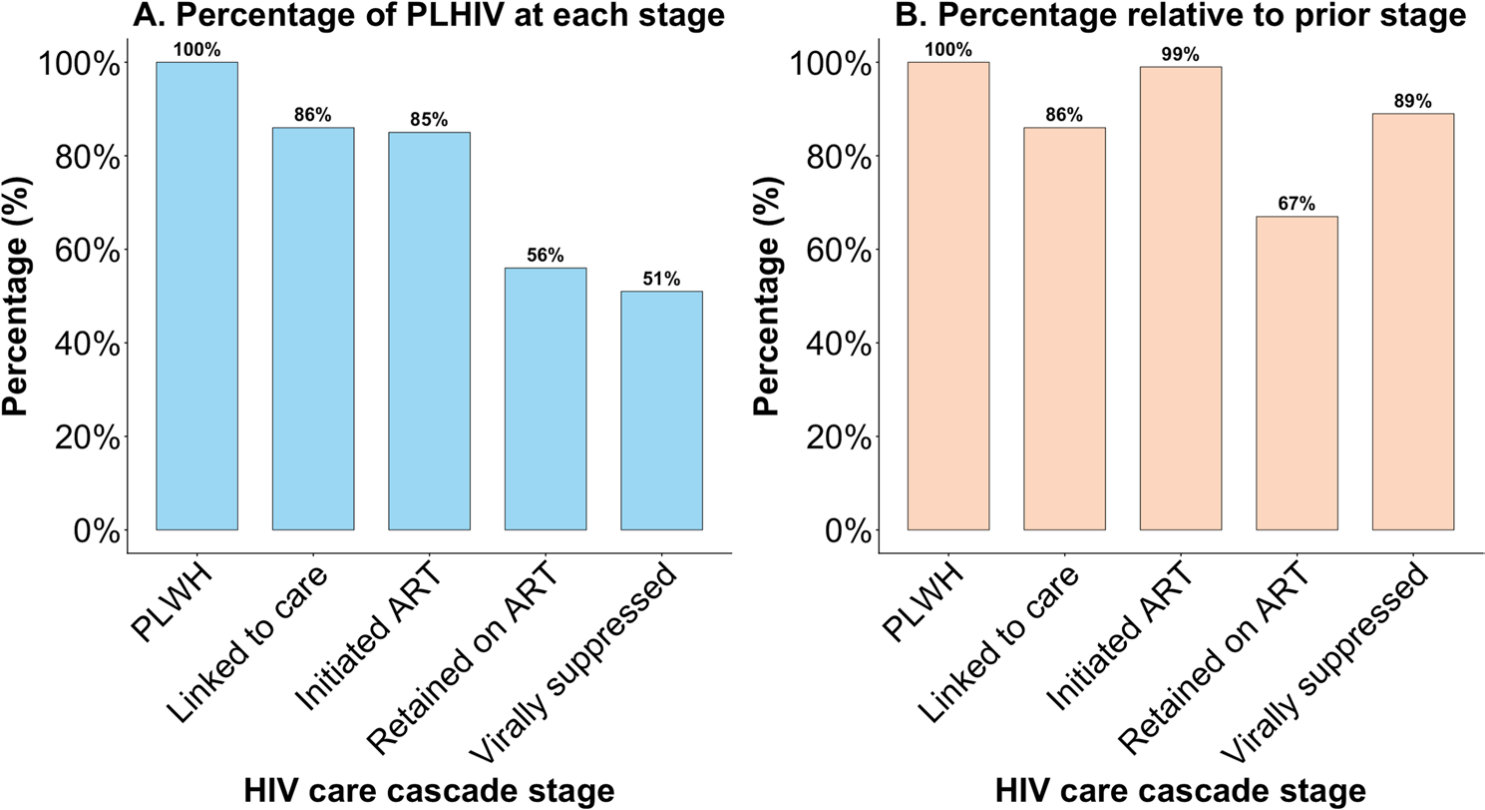
HIV care cascade among individuals observed in the Africa Health Research Institute Health and Demographic Surveillance between 2000 and 2024. Definitions for HIV care cascade stages in **A** are based on Supplementary Table S2 and HIV care cascade stages in **B** are based on Supplementary Table S3

The AUDP data enable construction of HIV care cascades for program monitoring based on the WHO HIV care cascade framework [24]. Supplementary Table S2 shows the HIV care cascade definitions used in the study. By the end of May 2024, 73,358 individuals aged 15 years and above had at least one dried blood test result in the HDSS. Of these, 21,058 (28.7%) adults were living with HIV. Of those with HIV positive results, 12,293(58.4%) resided in the HDSS at some point during 2022 and 2024. Among known HIV-positive persons residing in the HDSS in 2022 and 2024, 10,622 (86.4%) had ever sought HIV care in the public sector indicating successful linkage to care (TIER.Net ART visit, NHLS CD4 or viral load, AHRILink HIV visit or hospital admission). Of those, 10,492 (98.8%) had ever started ART (TIER.Net ART initiation, NHLS viral load, AHRILink ART visit or hospital admission) and 7,065 (66.5%) were currently on ART at their last HIV treatment visit (TIER.Net ART visit, NHLS viral load, AHRILink ART visit or hospital admission) and 2,402 (22.6%) were lost to follow up ( ≥365 days since their last documented HIV treatment visit or scheduled ART visit); of those currently on ART, 6,301 (89.2%) were virally suppressed (< 200 copies/mL) at their last viral load). Among all known HIV positive persons residing in the HDSS between 2022 and 2024, just 51.3% were currently on ART and had a documented VL < 200 copies/mL (Fig. 4). Supplementary Figure S2 shows these HIV care cascades stratified by age and sex with adolescents and young adults having poorer outcomes than older individuals based on Supplementary Table S2.

## Discussions

We deduplicated and linked HIV clinical, laboratory, and population-based HDSS records to create the AUDP using a graph-based probabilistic record linkage algorithm. Fully automated linkage, training and validation were efficient, reproducible, and scalable to other contexts.

The AUDP, integrating more than 23 years of clinical and population-based surveillance data, enables analyses of HIV care-seeking patterns and treatment outcomes within a well-documented, long-running population surveillance in rural KwaZulu-Natal [25] which has a high prevalence of infectious and non-communicable diseases [5, 10, 15, 26–30]. Linkage of these databases has the potential to accelerate HIV research and the translation of research into impactful interventions. For example, these data would allow assessment of the burden of opportunistic infections (e.g., tuberculosis) and comorbidities (e.g., cardiovascular diseases) among PLWH. Analysis of the CD4 count data at baseline or re-initiation can help us understand the prevalence and drivers of advanced HIV disease among patients initiating ART treatment. Mortality data from the HDSS could be used to pinpoint the stages within the HIV care and treatment cascade where mortality occurs.

National household surveys indicate that South Africa has made substantial progress toward UNAIDS’ 95-95-95 targets, with high levels of HIV status awareness (~ 90%), strong ART coverage among those diagnosed (~ 91%), and near-optimal viral suppression among individuals on treatment (~ 94%) [31]. In contrast, the HIV cascade in the AHRI-UDP reveals a more fragile care continuum. Although ART initiation among individuals who have ever accessed care is high (~ 99%), retention in care remains a major challenge: only two-thirds of known HIV-positive individuals were currently on ART, and more than one in five had been lost to follow-up. The higher rates of viremia among those currently on ART in our study – 11% vs. 6% nationally – further underscore the consequences of adherence gaps for those still in care. These discrepancies suggest two possible explanations: geographic heterogeneity and methodological differences. First, in the rural area covered by the AHRI-UDP, local health systems barriers may undermine long-term treatment continuity, despite high rates of linkage to care and ART initiation. Second, differences in data sources and definitions could explain the discrepancy. On the one hand, the AHRI-UDP excludes private sector care-seeking, which could underestimate numbers on ART. On the other hand, people with lapsed ART status may decline to participate in national surveys. National estimates based on programme monitoring databases are vulnerable to double counting. Both of these factors may over-estimate the number of patients on ART and underestimate attrition. The divergence of our findings from national estimates highlights the critical importance of further research to evaluate HIV programme performance at multiple epidemiological scales and in different data sources. National progress may mask localized weaknesses, and targeted interventions to improve patient retention and continuity of care will be essential to fully realize the population-level benefits of South Africa’s ART programme.

Our study builds on other health data linkage efforts in South Africa [4, 32, 33].

1. The Provincial Health Data Centre (PHDC) of Western Cape Province, South Africa, integrates administrative and clinical health data using a combination of deterministic and fuzzy marching approaches [32, 34]. PHDC uses a stepwise rule-based approach which uses a set of 48 rules. While 60% of the links fall into the “highly possible category”, duplicate merging relies on manual verification by clerks, and performance metrics have not been reported.
2. The Agincourt HDSS in Mpumalanga Province linked HDSS and local health facility data using fingerprint-based record pairs as the gold standard for deterministic and probabilistic approaches, achieving a fully automated linkage sensitivity of 83.6% and a PPV of 95.1%, which improved with 10% manual review [4]. The team also linked verbal autopsy data with the national civil registration system [33].
3. The NHLS National HIV Cohort was created by linking and deduplicating laboratory records from the NHLS of nearly all patients receiving HIV care in the public sector in South Africa [18, 19]. The record linkage approach, on which we build in this paper, yielded a sensitivity of 93.7% and a PPV of 98.6%. When patient identifiers are not available, databases can be linked on other common data elements. For example, TIER.Net and NHLS databases were linked based on CD4 and viral load test results yielding a sensitivity of 85.9% and a PPV of 96.8% [17].
4. The South African HIV Cancer Match (SAM) Study used a privacy-preserving probabilistic record linkage method to combine HIV-related data from the NHLS with cancer data from the National Cancer Registry (NCR) in South Africa [35–37]. Using the G-Link package, deduplicated NHLS data were linked to NCR data on encrypted names, gender, and date of birth. Linkage accuracy was validated for Gauteng Province using records with known South African IDs, resulting in a PPV of 98% and a sensitivity of 96%.
5. The Health Patient Registration System (HPRS) developed by South Africa’s National Department of Health and Centre for Scientific and Industrial Research (CSIR) has been implemented nationwide, with some deduplication and linkages with external databases [38]. However, validation statistics have not been systematically reported.
6. Finally, AHRI has engaged in prior record linkage efforts. Between 2004 and 2012, AHRI linked HDSS data with the Hlabisa HIV treatment programme, forming the ARTEMIS database [39]. However, funding for the programme ended in December 2012, and systematic linkage has not been maintained since then.

Building on these antecedents, our fully automated linkage algorithm achieved high accuracy through simultaneous linkage and deduplication of multiple data sources utilizing National IDs for training and validation. Our approach eliminates the need for costly, error-prone and subjective clerical review processes. Further, while some other linkage approaches have relied on attributes not typically captured in electronic health registers—such as a home address or another household member’s first name [4]— our approach achieved high accuracy while utilizing attributes routinely recorded across most health databases. Our approach has some limitations. First, non-representative validation sets can influence performance estimates. South African ID numbers were available for only 10.8% of records, and missingness may be associated with other attributes and how they are recorded. To correct for potential bias, we re-weighted the edges with ID numbers using the edge distribution for the whole database [23]. Second, as with any probabilistic linkage, the algorithm yielded small proportions of false negatives and false positives, which could lead to bias in analyses using these data. In a simulation study, we found that the bias due to these linkage errors is small for most analyses [40], however this is an area for further research. Finally, linkages of data with patient identifiers are constrained by government policies on data privacy and confidentiality, such as the Protection of Personal Information Act (POPIA) in South Africa. Owing to these restrictions, AHRI did not have access to the latest HIV treatment and laboratory data. Future efforts to update the AUDP will require record linkage approaches that can be implemented within this new regulatory environment, e.g. via linkage without patient identifiers [17], privacy-preserving strategies [35], and validated linkages conducted by health sector institutions such as NHLS [19] or district health offices. In conclusion, our study demonstrates that records from multiple data sources can be deduplicated and linked successfully with a high degree of accuracy in the context of a resource-poor, HIV-endemic region in rural South Africa. The resulting AUDP will help accelerate population-based and clinical research on HIV in rural South Africa.

## Implications for policy

Our successful linkage of HIV care records with population-based surveillance data demonstrates both the feasibility and strategic value of integrated data systems for monitoring the HIV epidemic in rural South Africa. These results highlight the need for policies that institutionalise secure, ethical, and routine data linkage across health information platforms. Strengthening data governance frameworks - particularly around access permissions, interoperability standards, and data quality assurance - will be critical to maximise data integration for health in South Africa. The AHRI Unified Data Platform offers a scalable model for how routinely collected clinical, laboratory, and surveillance data can be harmonised to generate near real-time insights, improve programmatic responsiveness, and support more efficient allocation of HIV resources.

The linked data also reveal important gaps in the local HIV care cascade that require targeted policy action. In contrast to national estimates showing high ART coverage and viral suppression among those aware of their status, the HDSS population exhibits markedly weaker retention, with high levels of loss to follow-up and lower viral suppression among those on ART. These findings underscore the need for policies that prioritise continuity of care, sustained engagement, and re-engagement in care, rather than focusing solely on diagnosis and ART initiation. Investments in differentiated service delivery models, strengthened patient tracing mechanisms, and community-led adherence support will be essential to address these gaps. Integrating real-time data systems across care platforms could further enable earlier identification of disengagement and facilitate rapid re-engagement. Closing these retention gaps is critical for achieving durable viral suppression at the population level and realising the full impact of South Africa’s HIV treatment programme.

## Supplementary Information

Supplementary Material 1. Table S1: Blocking strategies employed to create a master dataset submitted for scoring the edges, Table S2: HIV care cascade definitions used in the study, Table S3: HIV care cascade definitions used in the study, Figure S2: HIV care cascade1 by age and sex among resident individuals under surveillance in the Africa Health Research Institute Health and Demographic Surveillance System between 2022 and 2024.

## Data Availability

The de-identified data underlying this article were provided by Africa Health Research Institute (AHRI), South Africa’s National Department of Health (NDOH) and the National Health Laboratory Service (NHLS). While we received permission to use and analyse the data, we are not authorised to share it publicly. These institutions enforce strict confidentiality agreements and ethical approvals to protect patient privacy, prohibiting the sharing of the datasets with third parties without their prior approval. Data will be shared on request to the corresponding author with permission from the three parties. However, direct data requests can be made to AHRI ( [https://data.ahri.org/index.php/home](https://data.ahri.org/index.php/home) ), NDOH ( [https://nhrd.health.gov.za/](https://nhrd.health.gov.za) ) for TIER.Net data and NHLS ( [https://aarms.nhls.ac.za/](https://aarms.nhls.ac.za) ) for NHLS data.

## Abbreviations

AHRI: Africa Health Research Institute
NHLS: National Health Laboratory Service
TEIR.Net: Three Interlinked Electronic Registers
ART: Antiretroviral therapy
ID: Identification number
PPV: Positive predictive value
PII: Personal identifying information
