# Supplementary material for: Linkage of HIV treatment and population-based surveillance records in rural South Africa: the AHRI Unified Data Platform (AUDP)
Source: Arch Public Health. 2026 Feb 7;84:51. doi: 10.1186/s13690-026-01849-8 (PMC12977758; doi:10.1186/s13690-026-01849-8)
Supplement: Supplementary file 1 — Supplementary Material 1. Table S1: Blocking strategies employed to create a master dataset submitted for scoring the edges, Table S2: HIV care cascade definitions used in the study, Table S3: HIV care cascade definitions used in the study, Figure S2: HIV care cascade1 by age and sex among resident individuals under surveillance in the Africa Health Research Institute Health and Demographic Surveillance System between 2022 and 2024. [file 13690_2026_1849_MOESM1_ESM.docx]

**Supplementary Table S1:** Blocking strategies employed to create a master dataset submitted for scoring the edges

| **Blocking pass** | **1** | **2** | **3** | **4** | **5** | **6** | **7** | **8** | **9** | **10** | **11** | **12** | Total |
| --- | --- | --- | --- | --- | --- | --- | --- | --- | --- | --- | --- | --- | --- |
| **Attribute(s)** |  |  |  |  |  |  |  |  |  |  |  |  |  |
| First 4 letters of the first name | X | X |  |  |  |  |  |  |  |  |  |  |  |
| First 4 letters of surname | X |  | X |  |  |  |  |  |  |  |  |  |  |
| Last 4 letters of first name |  |  |  |  |  | X | X | X |  |  |  |  |  |
| Last 4 letters of surname |  |  |  |  |  | X | X |  | X |  |  |  |  |
| Last 1 letter of first name |  |  |  | X |  |  |  |  |  |  |  |  |  |
| Last 1 letter of surname |  |  |  |  | X |  |  |  |  |  |  |  |  |
| First name and second name inversion |  |  |  |  |  |  |  |  |  |  | X |  |  |
| First name and surname inversion |  |  |  |  |  |  |  |  |  | X |  |  |  |
| National ID number |  |  |  |  |  |  |  |  |  |  |  | X |  |
| Gender |  | X | X |  |  |  | X |  |  |  |  |  |  |
| Year of birth |  | X | X |  |  | X |  |  |  |  |  |  |  |
| Date of birth |  |  |  | X | X |  |  | X | X |  |  |  |  |
| Number of edges | 57,229,396 | 64,040,590 | 76,674,754 | 3,818,451 | 7,570,767 | 637,126 | 55,861,142 | 219,199 | 65,661 | 30,517 | 2,196,504 | 4,078 | 268,348,185 |

**Supplementary Figure S1**: Venn diagram of the linkage results for different data sources.

The figure shows the distribution (N, %) of 414,007 unique individuals across the databases. The interior percentages add up to 100%.


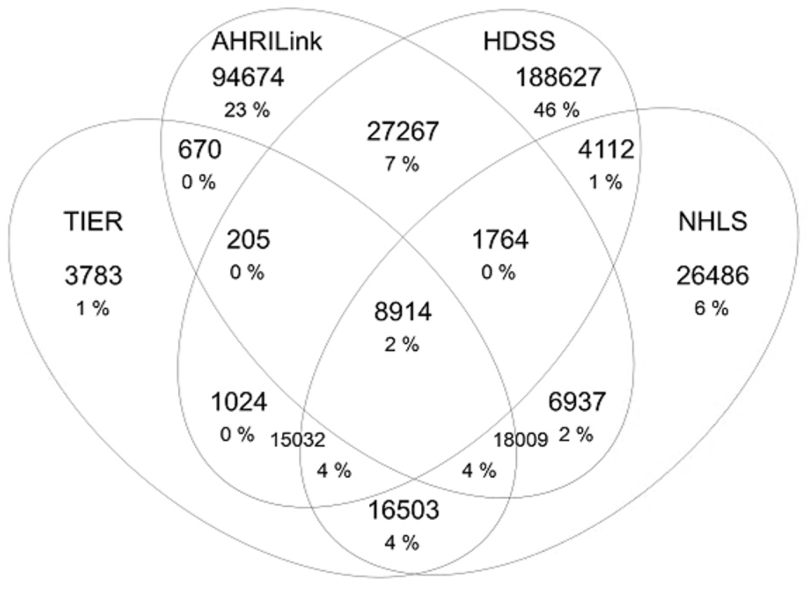


**Supplementary Table S2**: HIV care cascade definitions used in the study

| **HIV Care Cascade step** | **Definition** | **Numerator- denominator** |
| --- | --- | --- |
| People living with HIV(PLHIV) | Proportion of people testing positive for HIV through the dried blood spots collected from Africa Health Research Institute’s Health and Demographic Surveillance System | **Numerator:** All positive dried blood spots  **Denominator:** Adult residents tested for HIV |
| Diagnosed and aware of their HIV status | The proportion of people living with HIV who are aware of their HIV status  Dried blood spots results are not issued to research participants | Not applicable |
| Linked to care | The proportion of people living with HIV linked to care defined as having ART record in TIER.Net (recorded ART registration or initiation), CD4 count and viral load measurement from NHLS, reported ART initiation or follow clinic visit from AHRILink | **Numerator:** All individuals linked to care  **Denominator:** All adult residents tested HIV positive |
| Initiated ART | The proportion of people living with HIV who initiated ART defined as having recorded ART registration or initiation from TIER.Net, viral load measurement from NHLS, reported ART initiation or follow clinic visit from AHRILink (Hospital and clinics) | **Numerator:** All individuals with evidence of ART initiation  **Denominator:** All positive dried blood spots |
| Retained on ART | Proportion of people on sustained ART who initiated ART treatment defined as having a record in TIER.Net (ART clinic visit, ART medication), viral load measurement in NHLS and clinic visits from AHRILink (Hospital and clinics) | **Numerator:** All individuals with evidence of sustained ART treatment  **Denominator:** All positive dried blood spots |
| Virally suppressed | The proportion of people on ART treatment who have suppression of HIV defined as viral load of equal to or less than 200 copies/ml | **Numerator:** All individuals with evidence of sustained viral suppression  **Denominator:** All positive dried blood spots |

**Supplementary Table S3**: HIV care cascade definitions used in the study

| **HIV Care Cascade step** | **Definition** | **Numerator- denominator** |
| --- | --- | --- |
| People living with HIV(PLHIV) | Proportion of people testing positive for HIV through the dried blood spots collected from Africa Health Research Institute’s Health and Demographic Surveillance System | **Numerator:** All positive dried blood spots  **Denominator:** Adult residents tested for HIV |
| Diagnosed and aware of their HIV status | The proportion of people living with HIV who are aware of their HIV status  Dried blood spots results are not issued to research participants | Not applicable |
| Linked to care | The proportion of people living with HIV linked to care defined as having ART record in TIER.Net (recorded ART registration or initiation), CD4 count and viral load measurement from NHLS, reported ART initiation or follow clinic visit from AHRILink | **Numerator:** All individuals linked to care  **Denominator:** All adult residents tested HIV positive |
| Initiated ART | The proportion of people living with HIV who initiated ART defined as having recorded ART registration or initiation from TIER.Net, viral load measurement from NHLS, reported ART initiation or follow clinic visit from AHRILink (Hospital and clinics) | **Numerator:** All individuals with evidence of ART initiation  **Denominator:** All adult residents linked to HIV care |
| Retained on ART | Proportion of people on sustained ART who initiated ART treatment defined as having a record in TIER.Net (ART clinic visit, ART medication), viral load measurement in NHLS and clinic visits from AHRILink (Hospital and clinics) | **Numerator:** All individuals with evidence of sustained ART treatment  **Denominator:** All adult residents linked to HIV care |
| Virally suppressed | The proportion of people on ART treatment who have suppression of HIV defined as viral load of equal to or less than 200 copies/ml | **Numerator:** All individuals with evidence of sustained viral suppression  **Denominator:** All adult residents who started ART treatment |

**Supplementary Figure S2**: HIV care cascade^1^ by age and sex among resident individuals under surveillance in the Africa Health Research Institute Health and Demographic Surveillance System between 2022 and 2024

**
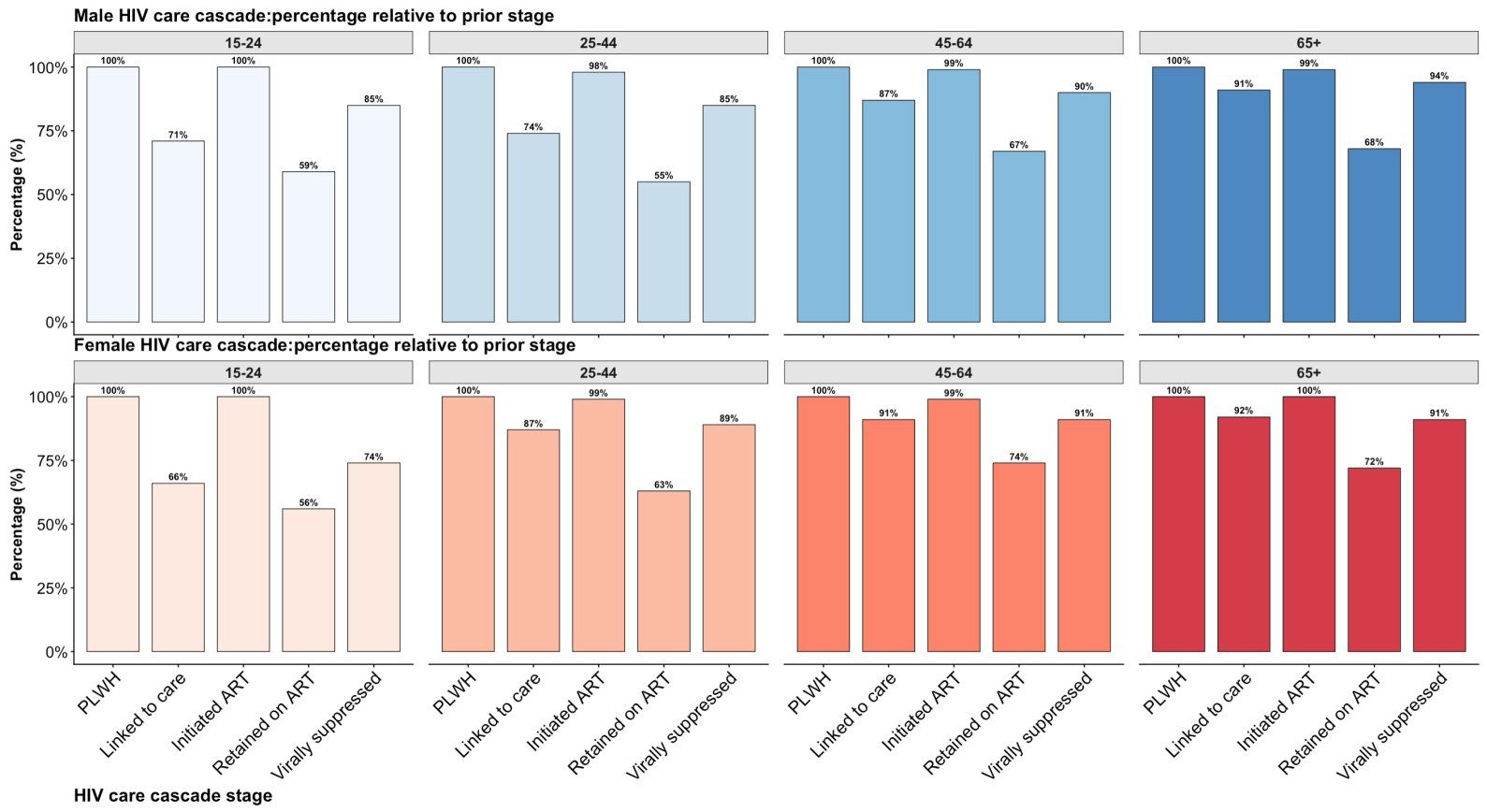
**
